# Comparison of Electrocautery and Scalpel Incisions in the Surgical Removal of Impacted Mandibular Third Molars: A Prospective Randomized Clinical Study

**DOI:** 10.7759/cureus.115730

**Published:** 2026-09-03

**Authors:** Dhaval B Rathod, Neha C Vyas, Anish R Tiwari, Darshan D Patel, Garv J Chaudhari, Raj D Madhani, Shrey D Patel, Vishva S Rajyaguru

**Affiliations:** 1 Oral and Maxillofacial Surgery, Ahmedabad Dental College and Hospital, Gandhinagar, IND

**Keywords:** blood loss, electrocautery, impacted mandibular third molar, scalpel blade, wound healing

## Abstract

Background

Surgical removal of impacted mandibular third molars is routinely performed using scalpel blade incisions; however, electrocautery has been proposed as an alternative due to its hemostatic advantage. Limited evidence exists comparing both techniques with respect to intraoperative efficiency and postoperative outcomes.

Aim

The main aim of this study is to compare electrocautery and scalpel blade incisions during surgical removal of impacted mandibular third molars in terms of operative time, blood loss, postoperative pain, facial swelling, trismus, and wound healing.

Materials and methods

A prospective split-mouth randomized study was conducted on 40 patients with bilateral impacted mandibular third molars. Each patient received an electrocautery incision on one side (study site) and a scalpel blade incision on the contralateral side (control site). The time taken for flap elevation and intraoperative blood loss were recorded. Postoperative pain was assessed using the Visual Analog Scale (VAS) at preoperative and 6, 12, 24, and 48 hours and at 7 days. Facial swelling and mouth opening were evaluated preoperatively and on postoperative days 3 and 7. Wound healing was assessed using the Landry healing index on days 7 and 14. Statistical analysis was performed using an independent t-test and repeated-measures ANOVA (p < 0.05).

Results

Electrocautery significantly reduced flap elevation time (6.65 ± 0.53 min vs. 7.31 ± 0.57 min; p < 0.001) and intraoperative blood loss (1.11 ± 0.42 mL vs. 1.66 ± 0.42 mL; p < 0.001). Pain scores peaked within 48 hours in both groups, with significantly lower pain in the electrocautery group at 24 hours (p = 0.047). No significant intergroup differences were observed for facial swelling or trismus at any time point (p > 0.05). Wound healing on day 7 was significantly better in the scalpel group (p < 0.001); however, by day 14, healing outcomes were comparable between the two groups (p = 0.562).

Conclusion

Electrocautery provides superior intraoperative efficiency by reducing surgical time and blood loss, with comparable postoperative outcomes to scalpel blade incisions. Although early wound healing may be delayed with electrocautery, long-term healing is unaffected. Electrocautery can be considered a safe and effective alternative for incisions in mandibular third molar surgery.

## Introduction

Surgical removal of impacted mandibular third molars is one of the most frequently performed minor oral surgical procedures in the field of oral and maxillofacial surgery. As reported by Babaji et al. [1] and Kalia et al. [2], this procedure is routinely carried out in clinical practice. The removal of impacted third molars is often indicated due to conditions such as pericoronitis, dental caries, periodontal disease, orthodontic considerations, or associated pathologies, including cysts and tumors, as highlighted by Goyal et al. [3].

The surgical procedure typically involves raising a mucoperiosteal flap, bone removal, tooth sectioning when necessary, and suturing to facilitate proper healing. Given that third molar surgery is invasive, meticulous surgical technique is essential to minimize tissue trauma and enhance postoperative recovery, as emphasized by Fatahi Vanani et al. [4].

Conventionally, incisions in third molar surgery are made using a Bard-Parker scalpel blade due to its precision, ease of use, and minimal damage to adjacent tissues. However, as noted by Jyotsna and Jha [5] and Sharma and Sachdeva [6], one of the major limitations of the scalpel is its inability to provide immediate hemostasis. Scalpel incisions involve mechanical disruption of soft tissues without coagulation of blood vessels, resulting in inevitable intraoperative bleeding. This bleeding can obscure the surgical field, increase the risk of inadvertent injury to surrounding structures, and prolong operative time, as reported by Ismail et al. [7].

Another concern associated with scalpel use is its psychological impact on patients. The visual perception of a blade may induce fear and anxiety, potentially leading to increased stress levels and a less favorable surgical experience, as observed by Asok et al. [8].

To overcome these limitations, alternative techniques for surgical incision have been explored. Among these, electrocautery has gained considerable attention due to its ability to produce precise incisions while simultaneously achieving hemostasis. Studies by Yilmaz et al. [9] and Kumar et al. [10] have demonstrated the effectiveness of electrocautery in soft tissue surgeries.

Electrocautery operates by passing high-frequency electrical current through tissues, generating heat that leads to cellular destruction and tissue separation. The resistance offered by biological tissues to this current results in effective cutting with simultaneous coagulation, as described by Gnanasekhar and Al-Duwairi [11]. One of its primary advantages is the reduction of intraoperative bleeding due to sealing of severed blood vessels, as highlighted by Nagargoje et al. [12] and Thakare et al. [13].

Additionally, electrocautery causes vaporization of microorganisms, thereby providing a relatively sterile surgical field. This may contribute to reduced postoperative complications such as pain, swelling, and trismus and promote uneventful healing, as discussed by Sharma and Sachdeva [6]. However, despite these advantages, some studies report no significant difference in healing outcomes between electrocautery and scalpel techniques, while others suggest that electrocautery may delay wound healing due to thermal damage at the incision margins, as noted by Kenneth et al. [14].

Given these contrasting findings, the present study aims to evaluate and compare the efficacy of electrocautery and scalpel incisions in the surgical removal of impacted mandibular third molars, with particular emphasis on operative time, intraoperative blood loss, postoperative sequelae such as pain, swelling, and trismus, and wound healing.

## Materials and methods

Study design, setting, and period

This prospective, randomized, split-mouth clinical trial was conducted in the Department of Oral and Maxillofacial Surgery at Ahmedabad Dental College and Hospital, Gujarat, India. The study was conducted from December 2022 to May 2025.

Patient recruitment

A total of 40 patients requiring bilateral surgical removal of impacted mandibular third molars were recruited from the outpatient department. Written informed consent was obtained from all participants prior to inclusion in the study.

Eligibility criteria

Patients aged 15-65 years with ASA I health status and bilaterally impacted mandibular third molars confirmed clinically and radiographically were included. Patients willing to comply with follow-up on the third and seventh postoperative days were enrolled. Cases associated with dentigerous cysts or other pathological lesions were excluded. Patients with cardiac pacemakers, immunodeficiency disorders, uncontrolled systemic diseases, anticoagulant therapy, or known drug allergies, as well as those who were uncooperative or mentally challenged, were also excluded.

Sample size calculation

The sample size was calculated using the following formula: $n = \frac{(Z_{\alpha/2} + Z_{1-\beta})^2 \times \sigma^2}{(m_1 - m_2)^2}$, where $Z_{\alpha/2} = 1.96$ at 95% confidence interval, $Z_{1-\beta} = 0.84$ at 80% power, *σ = 0.23*, and $m_1 = 5.3$, $m_2 = 5.4$. Based on the calculation, the final sample size was determined to be 40 patients with 80% power and a 5% level of significance using a two-sided paired design. The sample size estimation formula was adopted from the study by Fatahi Vanani et al. [4].

Preoperative assessment

All patients underwent a detailed preoperative evaluation as part of the study protocol, including case history, clinical and radiographic examination, and routine blood investigations comprising hemoglobin (Hb), bleeding time (BT), clotting time (CT), and random blood sugar (RBS).

Randomization

Each patient served as their own control in this split-mouth study. The two surgical sites were randomly allocated into study (electrocautery) and control (scalpel) groups using the lottery method. Only bilateral impacted mandibular third molars with the same Pederson Difficulty Index were selected to ensure comparable surgical difficulty [15].

Surgical procedure

Written procedure consent was obtained from all participants. All procedures were performed by the same surgeon under standard aseptic conditions. Painting and draping were performed using 5% povidone-iodine and spirit.

At the study site, an incision was made using an electrocautery unit (ART) with a T4 wire electrode in cut mode at an intensity of 2-4. At the control site, an incision was made using a Bard-Parker handle with a No. 15 blade.

In both groups, suction was avoided during incision and flap elevation, and blood was collected using preweighed dry gauze. A standard Ward’s incision was given, followed by mucoperiosteal flap reflection. Buccal osteotomy and odontectomy were performed when required using a micromotor handpiece and bur. This was followed by curettage, irrigation with sterile saline and betadine solution, and primary closure using interrupted 3-0 black braided silk sutures.

Data collection

Intraoperative parameters included the time taken for flap elevation, measured using a digital stopwatch, and blood loss, calculated as the difference between the dry and blood-soaked gauze weights, where 1 g was considered equivalent to 1 mL of blood loss. Pain was assessed using the Visual Analog Scale (VAS) preoperatively, before administration of local anesthesia, and postoperatively at 6, 12, 24, and 48 hours and on postoperative day 7. Facial swelling was measured using the modified Gabka and Matsumura method on postoperative days 3 and 7 [16]. Mouth opening was assessed as the maximum interincisal distance in millimeters, preoperatively and on postoperative days 3 and 7. Healing was evaluated using the modified Landry, Turnbull, and Howley healing index on postoperative days 7 and 14.

Postoperative care

All patients received standard postoperative instructions along with antibiotics (amoxicillin 500 mg or amoxicillin-clavulanic acid 625 mg twice daily for five days) and analgesics (paracetamol 500 mg with diclofenac sodium 50 mg).

Statistical analysis

Data were analyzed using IBM SPSS Statistics for Windows, Version 25 (Released 2017; IBM Corp., Armonk, NY, USA). Quantitative data were expressed as mean ± standard deviation. Paired t-test was used for comparison between study and control sites. Repeated measures ANOVA was used for comparison of parameters measured at multiple postoperative time intervals, followed by Bonferroni post hoc test for pairwise comparisons. A p-value of < 0.05 was considered statistically significant.

Ethical approval

Ethical approval was obtained from the Institutional Ethics Committee of Ahmedabad Dental College (IECADC), Gujarat, India, prior to commencement of the study (IECADC/0028/2022; dated 10/12/2022). Written informed consent was obtained from all participants before enrollment.

## Results

Statistical analysis was performed using the independent-samples t-test and ANOVA with IBM SPSS Statistics for Windows, Version 20 (Released 2011; IBM Corp., Armonk, NY, USA). A p-value of less than 0.05 was considered statistically significant.

A total of 40 participants were included in the study, of whom 60.0% (n = 24) were female and 40.0% (n = 16) were male (Table 1).

**Table 1:** Sex Distribution of Study Participants Distribution of participants by sex; data are presented as n and %; F = female, M = male; no statistical test was applied.

Sex	Frequency	Percent
F	24	60.0
M	16	40.0
Total	40	100.0

The age distribution showed that the majority of participants belonged to the 26-30-year age group (22.5%), followed by those aged 21-25 years (20.0%) and 15-20 years (17.5%). Participants aged 31-35 and 36-40 years each accounted for 12.5%, while those aged 41-45 and 46-50 years constituted 10.0% and 5.0%, respectively (Table 2).

**Table 2:** Age Distribution of Study Participants Age-wise distribution of participants (15-50 years). Data are presented as frequency (N) and percentage (%). Total sample size = 40. No statistical test was applied; the significance level was set at p < 0.05.

Age Group (years)	Frequency (N)	Percentage (%)
15-20	7	17.5
21-25	8	20.0
26-30	9	22.5
31-35	5	12.5
36-40	5	12.5
41-45	4	10.0
46-50	2	5.0
Total	40	100

The mean time required for flap elevation was significantly lower in the study group (6.65 ± 0.53 minutes) compared to the control group (7.31 ± 0.57 minutes), with a statistically significant difference (t = 5.436, p < 0.001) (Table 3).

**Table 3:** Time Taken for Flap Elevation (Minutes) Comparison of mean time taken for flap elevation between Study and Control groups. Data are presented as Mean ± SD. Intergroup comparison was performed using an unpaired t-test. The difference was statistically significant (p < 0.001).

Group	Mean (min)	Std. Deviation	Unpaired t	p-value
Study	6.65	0.53	5.436	<0.001
Control	7.31	0.57		

Similarly, the mean intraoperative blood loss was significantly reduced in the study group (1.11 ± 0.42 mL) compared with the control group (1.66 ± 0.42 mL), with a statistically significant difference (t = 5.966, p < 0.001) (Table 4).

**Table 4:** Blood Loss Comparison of intraoperative blood loss between groups. Data are expressed as mean ± SD and analyzed using an unpaired t-test (p < 0.05, statistically significant).

	Study		Control		Unpaired t	p-value
	Mean	Std. Deviation	Mean	Std. Deviation		
Blood Loss (in mL)	1.11	0.42	1.66	0.42	5.966	<0.001

Pain scores showed significant changes over time in both the study (F = 72.324, p < 0.001) and control (F = 83.215, p < 0.001) groups (Table 5).

**Table 5:** Pain Score Changes Over Time Intragroup comparison of pain scores over time. Data are expressed as mean ± SD and analyzed using repeated-measures ANOVA (p < 0.05, statistically significant).

Group	Time Interval	Mean ± SD
Study	Pain Pre Operative	0.58 ± 0.68
	Pain 6 Hrs	1.68 ± 0.86
	Pain 12 Hrs	2.50 ± 0.82
	Pain 24 Hrs	2.78 ± 0.66
	Pain 48 Hrs	2.60 ± 0.84
	Pain 7th Day	0.55 ± 0.68
Control	Pain Pre Operative	0.58 ± 0.68
	Pain 6 Hrs	1.73 ± 0.91
	Pain 12 Hrs	2.33 ± 0.73
	Pain 24 Hrs	3.10 ± 0.78
	Pain 48 Hrs	2.50 ± 0.75
	Pain 7th Day	0.68 ± 0.69

Bonferroni analysis demonstrated a significant increase in pain from preoperative levels to 6, 12, 24, and 48 hours in both groups, with no significant differences among the 12-48-hour time points and a return to baseline by day 7 (Table 6).

**Table 6:** Pain Post Hoc Analysis Pairwise comparison of pain scores using the Bonferroni post hoc test. Data are presented as p-values (p < 0.05, statistically significant).

Measure: Bonferroni					
Pairwise comparison (study group)			Pairwise comparison (control group)		
Comparison (time intervals)		Sig.	Comparison (time intervals)		Sig.
						Pre Op	6 Hr	<0.001	Pre Op	6 Hr	<0.001
12 Hr	<0.001	12 Hr	<0.001						
24 Hr	<0.001	24 Hr	<0.001						
48 Hr	<0.001	48 Hr	<0.001						
7 Day	1	7 Day	1						
6 Hr	Pre Op	<0.001	6 Hr	Pre Op	<0.001
	12 Hr	0.004		12 Hr	0.011
	24 Hr	<0.001		24 Hr	<0.001
	48 Hr	<0.001		48 Hr	0.004
	7 Day	<0.001		7 Day	<0.001
12 Hr	Pre Op	<0.001	12 Hr	Pre Op	<0.001
	6 Hr	0.004		6 Hr	0.011
	24 Hr	0.931		24 Hr	0.001
	48 Hr	1		48 Hr	1
	7 Day	<0.001		7 Day	<0.001
24 Hr	Pre Op	<0.001	24 Hr	Pre Op	<0.001
	6 Hr	<0.001		6 Hr	<0.001
	12 Hr	0.931		12 Hr	0.001
	48 Hr	1		48 Hr	0.011
	7 Day	<0.001		7 Day	<0.001
48 Hr	Pre Op	<0.001	48 Hr	Pre Op	<0.001
	2	<0.001		6 Hr	0.004
	12 Hr	1		12 Hr	1
	24 Hr	1		24 Hr	0.011
	7 Day	<0.001		7 Day	<0.001
7 Day	Pre Op	1	7 Day	Pre Op	1
	6 Hr	<0.001		6 Hr	<0.001
	12 Hr	<0.001		12 Hr	<0.001
	24 Hr	<0.001		24 Hr	<0.001
	48 Hr	<0.001		48 Hr	<0.001

Intergroup comparison revealed no significant differences at most time points; however, pain was significantly lower in the study group at 24 hours (2.78 ± 0.66 vs. 3.10 ± 0.78; p = 0.047) (Table 7).

**Table 7:** Intergroup Pain Comparison Comparison of pain scores between groups at different time intervals. Data are expressed as mean ± SD and analyzed using an unpaired t-test (p < 0.05, statistically significant).

	Study		Control		Unpaired t	p-value
	Mean	Std. Deviation	Mean	Std. Deviation		
Pain Pre Operative	0.58	0.68	0.58	0.68	0.102	1
Pain 6 Hrs	1.68	0.86	1.73	0.91	0.253	0.801
Pain 12 Hrs	2.50	0.82	2.33	0.73	1.011	0.315
Pain 24 Hrs	2.78	0.66	3.10	0.78	2.015	0.047
Pain 48 Hrs	2.60	0.84	2.50	0.75	0.561	0.577
Pain 7th Day	0.55	0.68	0.68	0.69	0.815	0.417

Overall, pain peaked within 48 hours and returned to baseline by day 7 in both groups, with improved pain control in the study group at 24 hours.

Facial swelling was assessed at preoperative, third-day, and seventh-day intervals using three measurements: tragus-corner of mouth (AC), tragus-pogonion (AD), and lateral canthus-gonion (BE). Repeated-measures ANOVA showed significant changes over time in both the study and control groups across all measurements (AC: F = 616.887 and 718.883; AD: F = 487.96 and 592.76; BE: F = 310.49 and 433.34; p < 0.001) (Tables 8-10).

**Table 8:** Facial Swelling (AC): Intragroup Comparison of Facial Swelling (Tragus-Corner of Mouth) Data are expressed as mean ± SD and analyzed using repeated-measures ANOVA (p < 0.05, statistically significant).

Group		Mean	Std. Deviation	F-value	p-value
Study	Facial Swelling (in mm) Pre Op AC	99.58	2.80	616.887	<0.01
	Facial Swelling (in mm) 3rd Day AC	105.00	2.95		
	Facial Swelling (in mm) 7th Day AC	100.98	2.87		
Control	Facial Swelling (in mm) Pre Op AC	99.55	2.70	718.883	<0.001
	Facial Swelling (in mm) 3rd Day AC	105.05	2.84		
	Facial Swelling (in mm) 7th Day AC	101.00	2.64		

**Table 9:** Facial Swelling (AD): Intragroup Comparison of Facial Swelling (Tragus-Pogonion) Data are expressed as mean ± SD and analyzed using repeated-measures ANOVA (p < 0.05, statistically significant).

Group		Mean	Std. Deviation	F-value	p-value
Study	Facial Swelling (in mm) Pre Op AD	104.58	3.06	487.96	<0.001
	Facial Swelling (in mm) 3rd Day AD	110.20	3.26		
	Facial Swelling (in mm) 7th Day AD	106.20	3.39		
Control	Facial Swelling (in mm) Pre Op AD	104.72	3.21	592.76	<0.001
	Facial Swelling (in mm) 3rd Day AD	110.40	3.23		
	Facial Swelling (in mm) 7th Day AD	106.23	3.33		

**Table 10:** Facial Swelling (BE): Intragroup Comparison of Facial Swelling (Exocanthion-Gonion) Data are expressed as mean ± SD and analyzed using repeated-measures ANOVA (p < 0.05, statistically significant).

Group		Mean	Std. Deviation	F-value	p-value
Study	Facial Swelling (in mm) Pre Op BE	96.53	2.54	310.49	<0.001
	Facial Swelling (in mm) 3rd Day BE	100.98	3.19		
	Facial Swelling (in mm) 7th Day BE	97.73	2.69		
Control	Facial Swelling (in mm) Pre Op BE	96.55	2.54	433.34	<0.001
	Facial Swelling (in mm) 3rd Day BE	101.15	2.92		
	Facial Swelling (in mm) 7th Day BE	97.80	2.67		

Bonferroni post hoc analysis demonstrated a significant increase in swelling from preoperative levels to day 3 and day 7 in both groups, with peak swelling at day 3 and a significant reduction by day 7, although values remained higher than baseline (p < 0.001) (Tables 11-13).

**Table 11:** Facial Swelling Post Hoc Analysis - Tragus to Corner of Mouth (AC) Pairwise comparison of facial swelling at the AC site across time points using the Bonferroni post hoc test. Data are presented as p-values, showing significant differences between preoperative, third-day, and seventh-day measurements (p < 0.05, statistically significant).

Group	Comparison of Time Intervals	Sig.
Study	Pre Op vs 3rd Day	<0.001
	Pre Op vs 7th Day	<0.001
	3rd Day vs Pre Op	<0.001
	3rd Day vs 7th Day	<0.001
	7th Day vs Pre Op	<0.001
	7th Day vs 3rd Day	<0.001
Control	Pre Op vs 3rd Day	<0.001
	Pre Op vs 7th Day	<0.001
	3rd Day vs Pre Op	<0.001
	3rd Day vs 7th Day	<0.001
	7th Day vs Pre Op	<0.001
	7th Day vs 3rd Day	<0.001

**Table 12:** Facial Swelling Post Hoc Analysis - Tragus to Pogonion (AD) Pairwise comparison of facial swelling at the AD site across time intervals using the Bonferroni test. Data are presented as p-values, indicating significant differences between all time points (p < 0.05, statistically significant).

Group	Comparison of Time Intervals	Sig.
Study	Pre Op vs 3rd Day	<0.001
	Pre Op vs 7th Day	<0.001
	3rd Day vs Pre Op	<0.001
	3rd Day vs 7th Day	<0.001
	7th Day vs Pre Op	<0.001
	7th Day vs 3rd Day	<0.001
Control	Pre Op vs 3rd Day	<0.001
	Pre Op vs 7th Day	<0.001
	3rd Day vs Pre Op	<0.001
	3rd Day vs 7th Day	<0.001
	7th Day vs Pre Op	<0.001
	7th Day vs 3rd Day	<0.001

**Table 13:** Facial Swelling Post Hoc Analysis - Exocanthion to Gonion (BE) Pairwise comparison of facial swelling at the BE site across time points using the Bonferroni test. Data are presented as p-values, showing significant differences between all intervals (p < 0.05).

Group	Comparison of Time Intervals	Sig.
Study	Pre Op vs 3rd Day	<0.001
	Pre Op vs 7th Day	<0.001
	3rd Day vs Pre Op	<0.001
	3rd Day vs 7th Day	<0.001
	7th Day vs Pre Op	<0.001
	7th Day vs 3rd Day	<0.001
Control	Pre Op vs 3rd Day	<0.001
	Pre Op vs 7th Day	<0.001
	3rd Day vs Pre Op	<0.001
	3rd Day vs 7th Day	<0.001
	7th Day vs Pre Op	<0.001
	7th Day vs 3rd Day	<0.001

Intergroup comparison revealed no statistically significant differences in facial swelling at any time point across all measurements, including preoperative (p > 0.8), day 3 (p > 0.7), and day 7 (p > 0.9), indicating comparable postoperative swelling between the groups (Tables 14-16).

**Table 14:** Intergroup Comparison of Facial Swelling - Tragus to Corner of Mouth (AC) Comparison of facial swelling between the study and control groups at the AC site. Data are expressed as mean ± SD and analyzed using an unpaired t-test, showing no statistically significant differences (p > 0.05).

	Study		Control		Unpaired t	p-value
	Mean	Std. Deviation	Mean	Std. Deviation		
Facial Swelling (in mm) Pre Op AC	99.58	2.80	99.55	2.70	0.041	0.968
Facial Swelling (in mm) 3rd Day AC	105.00	2.95	105.05	2.84	0.077	0.939
Facial Swelling (in mm) 7th Day AC	100.98	2.87	101.00	2.64	0.041	0.968

**Table 15:** Intergroup Comparison of Facial Swelling - Tragus to Pogonion (AD) Comparison of facial swelling between groups at the AD site. Data are expressed as mean ± SD and analyzed using an unpaired t-test, with no significant differences observed (p > 0.05).

	Study		Control		Unpaired t	p-value
	Mean	Std. Deviation	Mean	Std. Deviation		
Facial Swelling (in mm) Pre Op AD	104.58	3.06	104.72	3.21	0.214	0.831
Facial Swelling (in mm) 3rd Day AD	110.20	3.26	110.40	3.23	0.276	0.784
Facial Swelling (in mm) 7th Day AD	106.20	3.39	106.23	3.33	0.033	0.974

**Table 16:** Intergroup Comparison of Facial Swelling - Exocanthion to Gonion (BE) Comparison of facial swelling between the study and control groups at the BE site. Data are expressed as mean ± SD and analyzed using an unpaired t-test, showing no statistically significant differences (p > 0.05).

	Study		Control		Unpaired t	p-value
	Mean	Std. Deviation	Mean	Std. Deviation		
Facial Swelling (in mm) Pre Op BE	96.53	2.54	96.55	2.54	0.044	0.965
Facial Swelling (in mm) 3rd Day BE	100.98	3.19	101.15	2.92	0.256	0.799
Facial Swelling (in mm) 7th Day BE	97.73	2.69	97.80	2.67	0.125	0.901

Mouth opening (interincisal distance) showed significant changes over time in both the study (F = 1218.6, p < 0.001) and control (F = 963.2, p < 0.001) groups, indicating postoperative trismus (Table 17).

**Table 17:** Mouth Opening Over Time Intragroup comparison of mouth opening over time. Data are expressed as mean ± SD and analyzed using repeated-measures ANOVA (p < 0.05, statistically significant).

Group		Mean	Std. Deviation	F-value	p-value
Study	Mouth Opening (inter incisal distance) Pre Op	39.70	1.34	1218.6	<0.001
	Mouth Opening (inter incisal distance) 3rd Day	30.70	1.88		
	Mouth Opening (inter incisal distance) 7th Day	36.55	1.66		
Control	Mouth Opening (inter incisal distance) Pre Op	39.65	1.44	963.2	<0.001
	Mouth Opening (inter incisal distance) 3rd Day	30.53	1.66		
	Mouth Opening (inter incisal distance) 7th Day	36.68	1.49		

Bonferroni analysis revealed a significant reduction in mouth opening on day 3, followed by improvement on day 7, although values remained below preoperative levels (p < 0.001), with maximum trismus observed on day 3 (Table 18).

**Table 18:** Mouth Opening Post Hoc Analysis Pairwise comparison of mouth opening using the Bonferroni test. Data are presented as p-values (p < 0.05, statistically significant).

Group	Comparison of Time Intervals	Sig.
Study	Pre Op vs 3rd Day	<0.001
	Pre Op vs 7th Day	<0.001
	3rd Day vs Pre Op	<0.001
	3rd Day vs 7th Day	<0.001
	7th Day vs Pre Op	<0.001
	7th Day vs 3rd Day	<0.001
Control	Pre Op vs 3rd Day	<0.001
	Pre Op vs 7th Day	<0.001
	3rd Day vs Pre Op	<0.001
	3rd Day vs 7th Day	<0.001
	7th Day vs Pre Op	<0.001
	7th Day vs 3rd Day	<0.001

Intergroup comparison showed no statistically significant differences at any time point (p > 0.6), indicating a similar pattern of trismus and recovery in both groups (Table 19).

**Table 19:** Intergroup Mouth Opening Comparison Comparison of mouth opening between groups. Data are expressed as mean ± SD and analyzed using an unpaired t-test (p < 0.05, statistically significant).

	Study		Control		Unpaired t	p-value
	Mean	Std. Deviation	Mean	Std. Deviation		
Mouth Opening (inter incisal distance) Pre Op	39.70	1.34	39.65	1.44	0.16	0.873
Mouth Opening (inter incisal distance) 3rd Day	30.70	1.88	30.53	1.66	0.44	0.661
Mouth Opening (inter incisal distance) 7th Day	36.55	1.66	36.68	1.49	0.354	0.724

Healing of the surgical site improved significantly over time in both the study and control groups (p < 0.001) (Table 20).

**Table 20:** Healing Over Time Intragroup comparison of healing scores. Data are expressed as mean ± SD and analyzed using a paired t-test (p < 0.05, statistically significant).

Group		Mean	Std. Deviation	t	p-value
Study	Healing of surgical site 7th Day	2.63	0.59	17.406	<0.001
	Healing of surgical site 14th Day	4.15	0.58		
Control	Healing of surgical site 7th Day	3.15	0.36	14.036	<0.001
	Healing of surgical site 14th Day	4.08	0.57		

On day 7, healing was significantly better in the control group (3.15 ± 0.36) compared to the study group (2.63 ± 0.59) (t = 4.824, p < 0.001). However, by day 14, no statistically significant difference was observed between the study (4.15 ± 0.58) and control groups (4.08 ± 0.57) (p = 0.562) (Table 21).

**Table 21:** Intergroup Healing Comparison Comparison of healing between groups. Data are expressed as mean ± SD and analyzed using an unpaired t-test (p < 0.05, statistically significant).

	Study		Control		Unpaired t	p-value
	Mean	Std. Deviation	Mean	Std. Deviation		
Healing of surgical site 7th Day	2.63	0.59	3.15	0.36	4.824	<0.001
Healing of surgical site 14th Day	4.15	0.58	4.08	0.57	0.592	0.562

These findings indicate delayed early healing in the study group, with comparable healing outcomes in both groups by day 14.

## Discussion

This study offers a comprehensive comparative evaluation of electrocautery and scalpel techniques for incisions made during surgical removal of impacted mandibular third molars, focusing on both intraoperative efficiency and postoperative healing outcomes. The results reflect a growing interest in incorporating modern surgical technologies, such as electrocautery, to improve operative parameters while maintaining acceptable healing and patient comfort levels.

The study sample was representative of a typical young adult population undergoing third molar surgery, with a higher proportion of females (60%) and most participants falling within the 21-30-year age bracket. A split-mouth design was employed, with each patient serving as their own control, effectively reducing variability due to patient-related factors. For each selected patient, the two surgical sites were randomly assigned to different groups using the lottery method: the study site received an intraoral incision using electrocautery, while the control site received an incision using a No. 15 scalpel blade. All surgeries were performed by a single experienced surgeon using a standardized protocol, thereby ensuring consistency across procedures and improving the reliability of the findings.

Electrocautery significantly reduced the time taken for flap elevation (6.65 ± 0.53 minutes vs. 7.31 ± 0.57 minutes, p < 0.001). This likely reflects the dual action of cutting and coagulation provided by electrocautery, which coagulates blood vessels at the time of incision and provides a clear surgical field, allowing for more efficient dissection and improved visibility and ultimately reducing incision time. In terms of blood loss, electrocautery was also superior, with significantly lower mean blood loss (1.11 ± 0.42 mL) compared to scalpel incisions (1.66 ± 0.42 mL, p < 0.001). These findings align with previous literature reported by Sharma and Sachdeva [6], Nagargoje et al. [12], and Ismail et al. [7], which similarly demonstrated decreased bleeding and shorter operative times with electrocautery.

Pain, a primary concern in oral surgery, peaked for both groups within the first 24 hours and decreased progressively over the week. Interestingly, pain scores at 24 hours were significantly lower in the electrocautery group (p = 0.047), potentially due to reduced nerve stimulation and immediate sealing of nerve endings through coagulation. This observation is consistent with studies by Goyal et al. [3], which suggested that electrocautery may offer transient analgesic benefits. However, no significant differences in pain levels were noted at other time points, indicating that the overall pain trajectories were similar.

Postoperative swelling followed a predictable course in both groups, peaking on the third postoperative day and reducing by the seventh day. All three linear facial measurements (AC, AD, and BE) showed significant within-group changes over time, but no statistically significant intergroup differences were detected. These findings suggest that facial edema is more closely related to the surgical trauma itself rather than the incision modality used. This conclusion is in line with the findings of Fatahi Vanani et al. [4], who also reported no significant difference in swelling between electrosurgery and scalpel incisions.

Similarly, the pattern of trismus, measured using interincisal distance, showed a postoperative decrease on day 3 followed by partial recovery by day 7. No statistically significant differences were observed between the two groups, reinforcing that trismus is more likely a consequence of surgical manipulation and inflammation rather than the specific incision method employed.

The most notable difference between the two techniques was observed in the rate of wound healing. On the seventh postoperative day, healing was significantly better in the scalpel blade group compared to the electrocautery group (p < 0.001), likely due to the thermal tissue damage induced by cauterization. These findings corroborate earlier histological studies by Kenneth et al. [14] and Liboon et al. [17], which demonstrated deeper tissue necrosis and delayed epithelialization associated with electrocautery incisions. However, by the 14th postoperative day, the healing scores had equalized between groups (p = 0.562), suggesting that long-term healing is not adversely affected by electrocautery. Similar observations were reported by Rathofer et al. [18] and Goyal et al. [3], who found comparable long-term healing outcomes despite delayed early healing in electrosurgical techniques.

The findings of the present study are in broad agreement with existing literature evaluating electrocautery in oral and general surgical procedures. Studies by Sharma and Sachdeva [6], Yadav et al. [19], and Elbohoty et al. [20] consistently demonstrated reduced intraoperative blood loss and shorter operative duration with electrocautery. Likewise, the delayed early healing observed in the present study is consistent with findings reported by Yilmaz et al. [9] and Uludag et al. [21], who described increased early tissue inflammation and thermal injury associated with electrocautery, although these effects were temporary and did not affect long-term healing.

Additionally, the transient pain reduction observed at 24 hours aligns with the findings of Nagargoje et al. [12] and Asok et al. [8], who emphasized the potential analgesic benefits of thermal surgical techniques in the immediate postoperative period. Conversely, Kashkouli et al. [22] and Hasar et al. [23] cautioned against excessive tissue charring and delayed epithelialization, emphasizing the need for controlled energy settings and precise technique while using electrocautery.

From a clinical standpoint, electrocautery appears to offer several practical advantages, including reduced operative time, improved hemostasis, and potential early postoperative pain relief. However, clinicians should remain cautious regarding the possibility of delayed early healing and thermal injury to adjacent tissues. Although these effects are temporary, they may be clinically relevant in cases where rapid tissue healing or superior cosmetic outcomes are essential.

Limitations of the study

The present study had certain limitations, including a relatively small sample size and a short follow-up period of 14 days, which limited long-term evaluation of healing outcomes. Additionally, histological evaluation of wound healing was not performed. Further studies with larger sample sizes and longer follow-up periods are recommended to validate the findings.

## Conclusions

Based on all the observations and statistical analyses, we can conclude that electrocautery offers certain intraoperative advantages over the conventional scalpel technique in the surgical removal of impacted mandibular third molars. Specifically, electrocautery significantly reduces operative time and intraoperative bleeding, which can contribute to improved surgical efficiency and visibility. However, electrocautery may cause a slight delay in early healing; this difference resolves by the 14th postoperative day, with no significant long-term impact. Additionally, postoperative parameters such as pain, swelling, mouth opening, and healing showed no statistically significant differences between the two techniques by the end of the observation period. Thus, both electrocautery and scalpel blade techniques are effective and safe, and the choice of incision method can be guided by the surgeon’s preference, clinical setting, and need for intraoperative hemostasis.

Electrocautery may be particularly beneficial in patients in whom minimizing bleeding and improving surgical field clarity are prioritized. However, a limitation of this study was the limited sample size; further studies with larger sample sizes are needed to obtain more conclusive evidence regarding the efficacy of the study material.
